# Current State of Potential Mechanisms Supporting Low Intensity Focused Ultrasound for Neuromodulation

**DOI:** 10.3389/fnhum.2022.872639

**Published:** 2022-04-25

**Authors:** John Dell'Italia, Joseph L. Sanguinetti, Martin M. Monti, Alexander Bystritsky, Nicco Reggente

**Affiliations:** ^1^Institute for Advanced Consciousness Studies, Santa Monica, CA, United States; ^2^Department of Psychology, University of Arizona, Tuscon, AZ, United States; ^3^Department of Psychology, University of New Mexico, Albuquerque, NM, United States; ^4^Department of Psychology, University of California, Los Angeles, Los Angeles, CA, United States; ^5^Brain Injury Research Center, Department of Neurosurgery, David Geffen School of Medicine at University of California, Los Angeles, Los Angeles, CA, United States; ^6^Tiny Blue Dot Foundation, Santa Monica, CA, United States

**Keywords:** non-invasive brain stimulation, neuromodulation, low intensity focused ultrasound, focused ultrasound stimulation, transcranial focused ultrasound

## Abstract

Low intensity focused ultrasound (LIFU) has been gaining traction as a non-invasive neuromodulation technology due to its superior spatial specificity relative to transcranial electrical/magnetic stimulation. Despite a growing literature of LIFU-induced behavioral modifications, the mechanisms of action supporting LIFU's parameter-dependent excitatory and suppressive effects are not fully understood. This review provides a comprehensive introduction to the underlying mechanics of both acoustic energy and neuronal membranes, defining the primary variables for a subsequent review of the field's proposed mechanisms supporting LIFU's neuromodulatory effects. An exhaustive review of the empirical literature was also conducted and studies were grouped based on the sonication parameters used and behavioral effects observed, with the goal of linking empirical findings to the proposed theoretical mechanisms and evaluating which model best fits the existing data. A neuronal intramembrane cavitation excitation model, which accounts for differential effects as a function of cell-type, emerged as a possible explanation for the range of excitatory effects found in the literature. The suppressive and other findings need additional theoretical mechanisms and these theoretical mechanisms need to have established relationships to sonication parameters.

## 1. Introduction

Non-invasive brain stimulation (NIBS) has been limited due to a lack of technology that matches the spatial precision of invasive techniques. The most widely-employed NIBS techniques across empirical research and clinical practices are transcranial electrical stimulation (TES) and transcranial magnetic stimulation (TMS). TES, which delivers current between electrodes placed along the scalp to purportedly produce weak electrical fields in the brain, inherently permits for the diffuse spread of current over large swaths of non-targeted regions of cortex (Datta et al., 2009; Reinhart and Woodman, 2015). TMS, which leverages an alternating magnetic field to electromagnetically induce electrical current in the brain, has been shown to have as low as 5 cm^2^ and as high as 273 cm^2^ of tangential spread—a loss of focality that worsens as a function of cortical depth (Deng et al., 2013), preventing sub-cortical stimulation. While newer multichannel TES approaches (Ruffini et al., 2018; Mencarelli et al., 2020) partially alleviate these spatial resolution concerns, Focused Ultrasound (FU) is a promising alternative with superior spatial resolution and penetration depth compared to TES and TMS. FU has grown in popularity due partially to its ability to leverage magnetic-resonance(MR)images and modeling to target sub-cortical regions without damaging intervening tissue. FU is an emerging field; a PubMed search with terms “ultrasound AND neuromodulation” reveals a 100% year-over-year growth since 2015. Despite this growing corpus, LIFU's mechanism(s) of action are not fully understood and the development of a reliable, parameter-dependent handbook for inducing excitatory and suppressive neuronal effects is still in its infancy. This review is structured to provide a comprehensive introduction to the underlying mechanics of LIFU, defining the primary variables and parameter spaces for a subsequent review of the proposed mechanisms supporting LIFU's excitatory vs. inhibitory neuromodulatory effects. Subsequently, existing empirical literature is grouped as a function of sonication parameters used and behavioral effects observed. Finally, we provide a novel synthesis of empirical findings in context of the proposed theoretical mechanisms, evaluating which model best fits the existing data in hopes of shedding light on which parameter spaces should be further explored as well as providing implications for safe use and potential long-term effects.

## 2. Introduction to Ultrasound Mechanics

**Figure 1:**
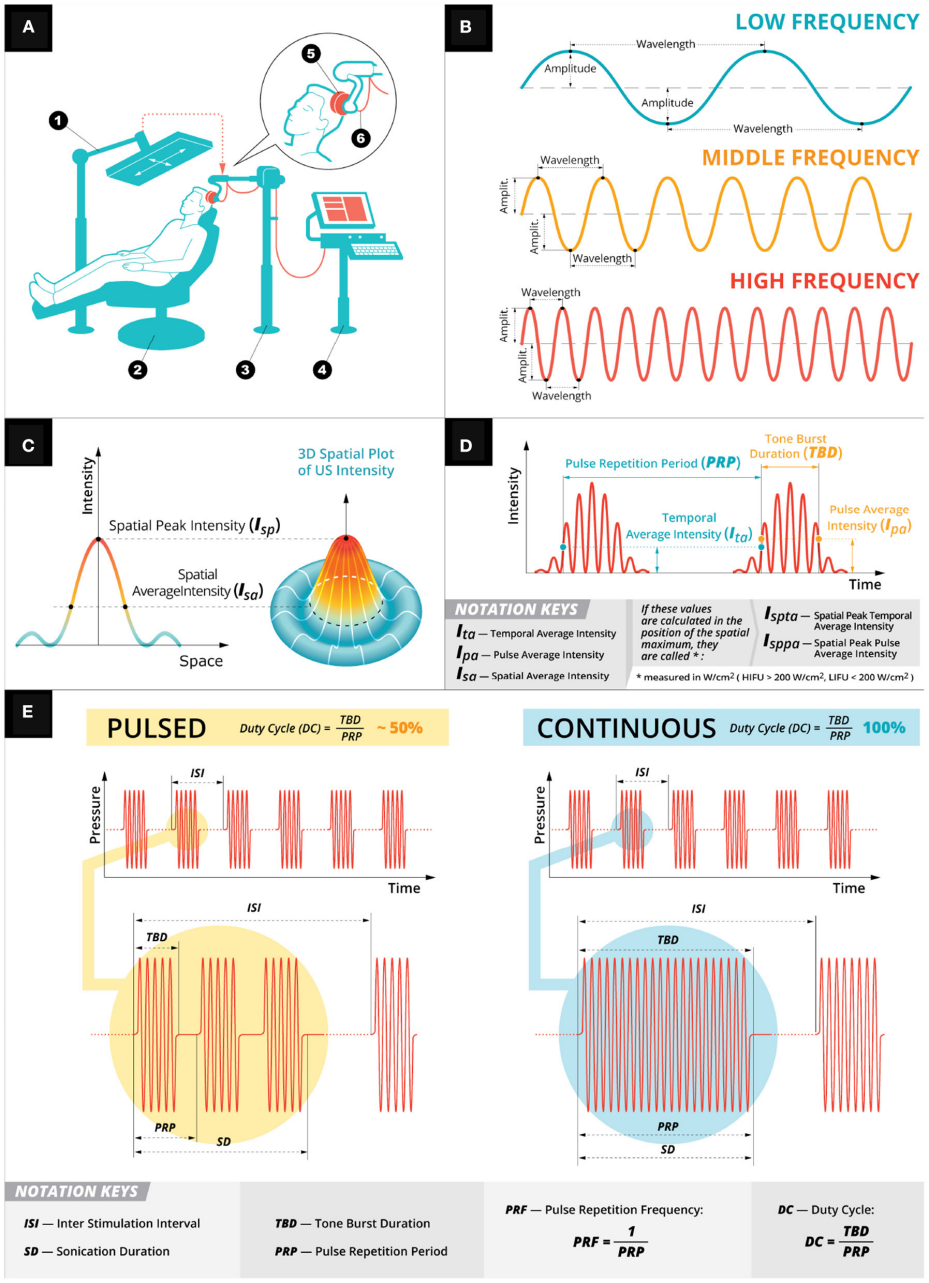
Low intensity focused ultrasound general principles. **(A)** A depiction of a typical LIFU experimental setup. A participant is seated (2) with an US device (5) firmly pressed against their head held in place by an arm (3). The US device is controlled by a computer system (4) and targeted using infrared system (1). **(B)** Depiction of the mechanical wave properties (amplitude, wavelength, and frequency) used in US stimulation. **(C)** Spatial intensities of the mechanical wave. **(D)** Temporal intensities of the mechanical wave. **(E)** Two exemplary pulsation schemes: pulsed (in yellow) and continuous (in teal). Both the pulsing schemes have a customizable sonication duration with inter stimulation interval with the DC parameter (i.e., the ratio of tone burst duration over pulse repetition period) determining the pulsing scheme.

Ultrasound (US) generates acoustic waves with characteristic properties (see Figure 1B) of wavelength, amplitude, and frequency.There exists a broad band of US frequency (100 kHz–100 MHz); an increase in frequency decreases the area affected by the focal point of the acoustic waves (i.e., increases spatial precision). Unfocused US for imaging or diagnostic tests is typically between 2 and 15 MHz (Shung, 2009). Focused US (FUS) for neuromodulation is typically below 2 MHz. High intensity focused ultrasound (HIFU) is used to ablate tissue. Low intensity focused ultrasound (LIFU) is used to generate temporary neuromodulation.

HIFU and LIFU use a transducer to deliver an acoustic wave of mechanical energy to a target stimulation site (Figure 1A) (Cline et al., 1992; Lipsman et al., 2013; Magnin et al., 2015). The transducer converts alternating current into pressure waves, controlled by a wave generator with sonication parameters that affect the stimulation outcome and efficacy: center frequency [f_*c*_], sonication duration (SD), interstimulus interval (ISI), tone burst duration (TBD), pulse repetition period (PRP), pulse repetition frequency (PRF), and duty cycle (DC) as well as pulsed vs. continuous schemes. SD is the total duration of each FUS's acoustic wave and the ISI is the time between each SD (Figure 1E). The SD is determined by the TBD (i.e., total duration of acoustic wave; Figure 1E) and the time between tone bursts (PRP; Figure 1E). PRP is the inverse of PRF—the number of pulses delivered in a 1 s period of time). DC is the ratio of TBD over PRP—a percentage of the sonication duration that acoustic waves are being delivered, which determines the pulsing schemes of either pulsed (i.e., DC < 100%) or continuous (i.e., no gaps between tone bursts, which is DC = 100%; Figure 1E). Finally, intensity is calculated based on either the temporal average (I_*ta*_; i.e., the temporal average across PRP; Figure 1D) and/or pulse average (I_*pa*_; i.e., pulse average across TBD—see Figure 1D). The intensity varies spatially (Figure 1C) depending on the focal point of stimulation and can be calculated at the spatial peak for either I_*ta*_ (I_*spta*_) or I_*pa*_ (I_*sppa*_). These intensities are calculated using a hydrophone, which can measure the mechanical pressure in liquids and convert those voltage recordings into acoustic pressure measurements. Alternatively, these voltage recordings can be converted into intensities using a pulse intensity integral measured in watts per centimeter squared (i.e., W/cm^2^). When these intensities are calculated using a hydrophone in a degassed water tank (Retz et al., 2017), the intensity is not derated (I_*spta*.0_) or (I_*sppa*.0_) for any tissue attenuation, which can be assumed as a uniform 0.3 dB/cm-MHz derating and reported as a derated (I_*spta*.3_) or (I_*sppa*.3_) (Schafer et al., 2020). However, this uniform attenuation does not account for the skull, which can attenuate between 50% and 80% (Mueller et al., 2017; Legon et al., 2018a; Phipps et al., 2019) of the pressure. This attenuation is partly due to a mode conversion that occurs when the wave interacts with the skull at an incidence angle not equal to the reflected angle transforming the longitudinal wave—a wave propagation direction parallel to particle motion direction originating from the transducer—into a shear wave—a wave propagation direction perpendicular to particle motion direction. Attenuation can also be attributed to reflection, scattering (Fry and Barger, 1978), and, to a lesser extent, bone absorption (Pinton et al., 2012; Phipps et al., 2019). To reconcile this energy attenuation, skull samples (Phipps et al., 2019), or computer simulations (Pinton et al., 2010; Mueller et al., 2017; Legon et al., 2018a,b) are used to estimate the intensity of the acoustic waves in the underlying neural tissue.

In HIFU, the relationship between sonication parameters tissue ablation is widely understood to stem from thermal sources (i.e., heat from the mechanical wave interacting with the tissue) (Haar, 2010). Conversely, the mechanisms of action for neuromodulation are not as well-established. In this review, two sets of literature are explored: theoretical mechanisms of mechanical energy interactions with neural tissue (Krasovitski et al., 2011; Plaksin et al., 2014, 2016; Jerusalem et al., 2019) and empirical LIFU studies grouped based on behavioral, electrophysiology, and neuroimaging outcomes. We performed a literature review of published studies from 2008 through July, 2021 using the following search criteria: ultrasound, low intensity focused ultrasound, low intesnsity focused ultrasound pulsation, LIFU, LIFUP, and neuromodulation. The goal of this review is understanding parameter-specific outcomes in the context of proposed theoretical mechanisms.

## 3. Mechanisms for Neuromodulation *via* US

### 3.1. Electrophysiological-Mechanical Coupling

**Figure 2:**
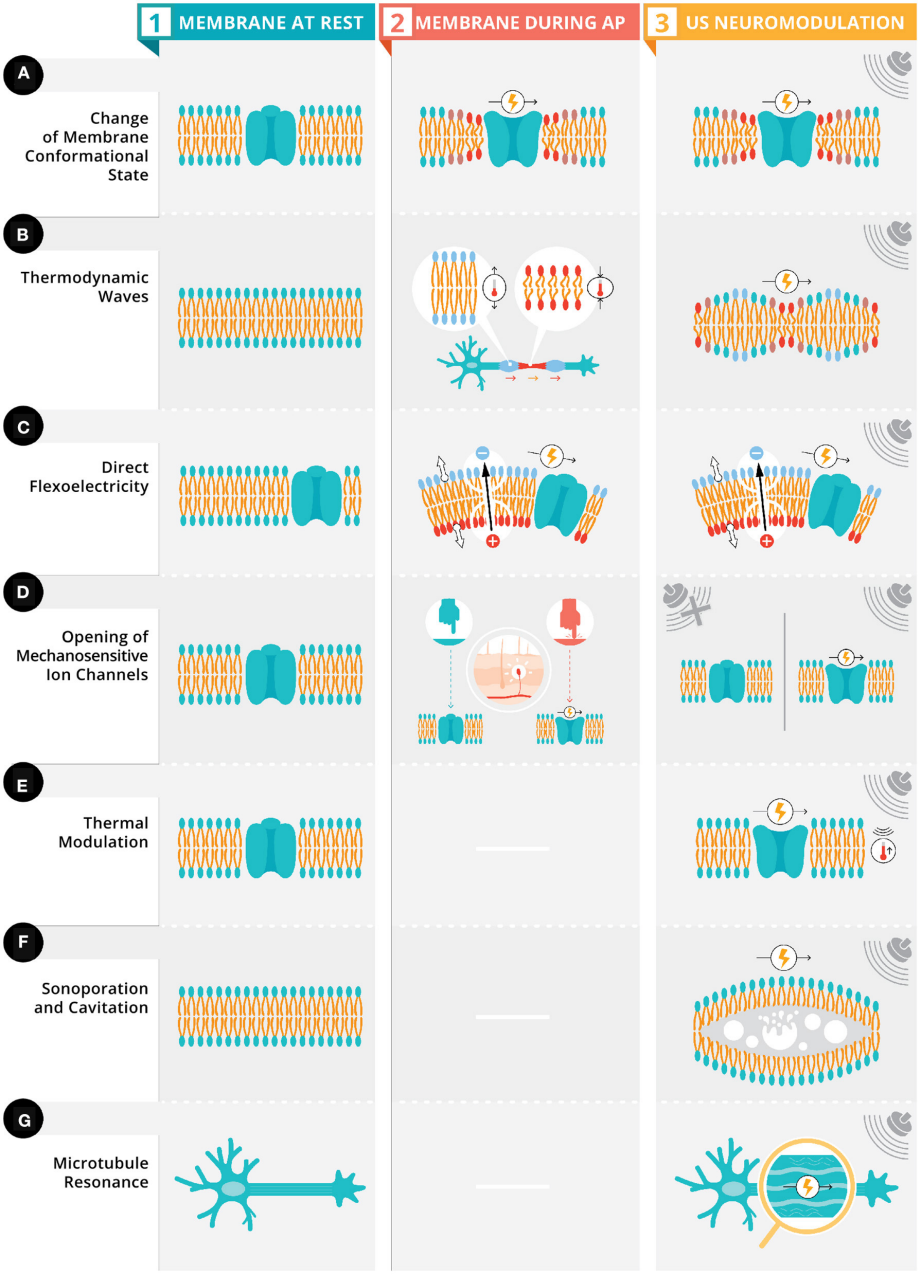
Proposed ultrasonic stimulation's mechanisms for neuromodulation. Depicted in column 1 are six neuronal membranes (four with an ion channel [rows **A,C,D,E**] and two neuronal membranes [rows **B,F**] with polar lipid bilayer) and a neuron with the microtubules highlighted (row **G**). Depicted in column 2, these membranes have four types of electrophysiological-mechanical coupling during an action potential: change in membrane conformation state, thermodynamic waves, direct flexoelectricity, and opening of mechanosensitive ion channels (see Section above). Column 3 depicts these same four electrophysiological-mechanical coupling during US stimulation along with three other possible mechanisms of US's neuromodulation: thermal modulation, sonoporation and cavitation, and microtubule resonance (see Section above).

**Figure 3:**
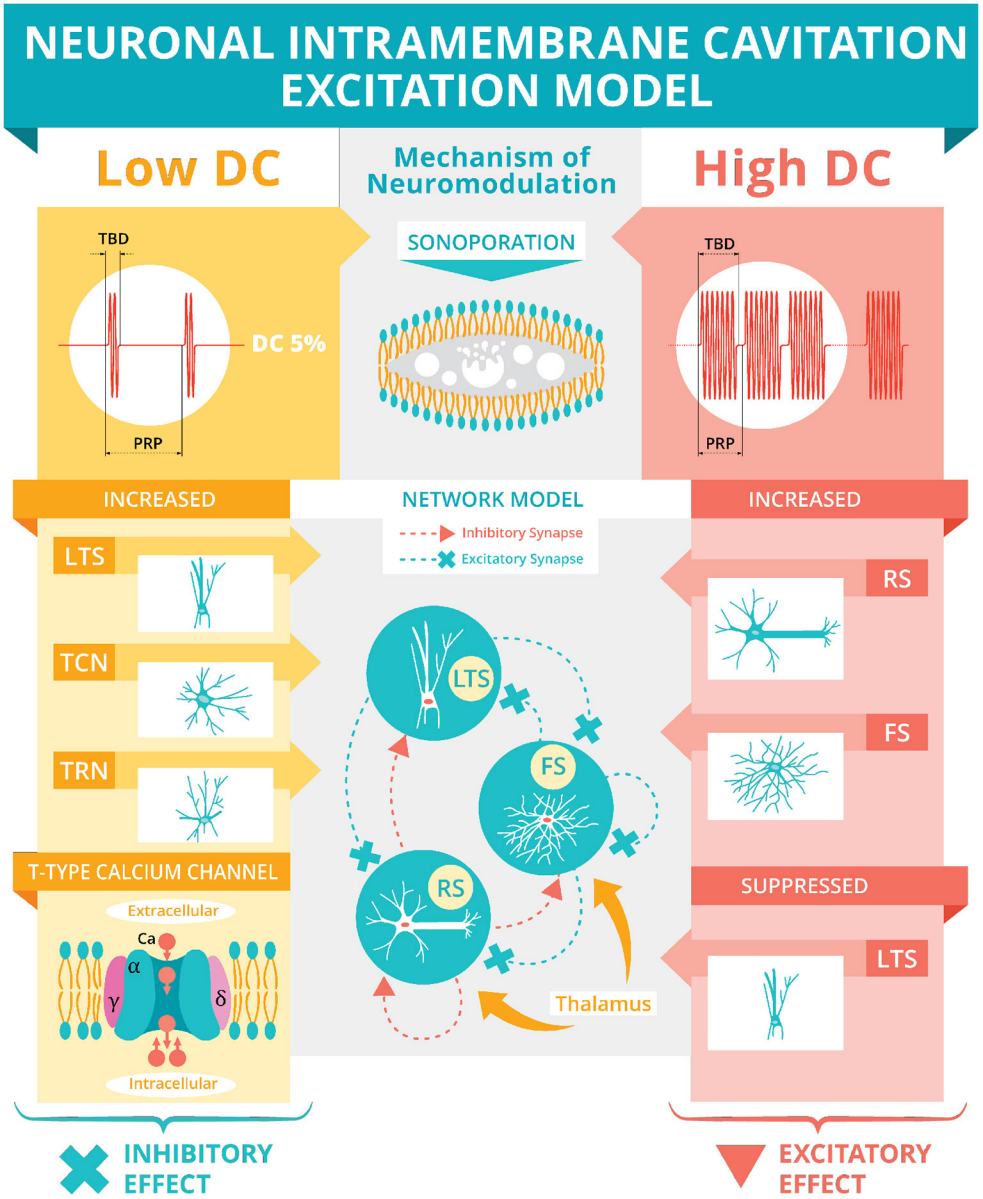
Neuronal intramembrane cavitation excitation model. Plaksin et al. (2014, 2016) proposed the NICE model hypothesizing sonoporation (see Section above and Figure 2F) as US's mechanism of neuromodulation. The US's DC (see Figure 1E) determines the polarity of neuromodulation. A low DC (i.e., below 5%) during a stimulation's off-periods will preferentially activate thalamic reticular neurons (TRN), thalamocortical neurons (TCN), and low-threshold spiking (LTS) interneurons *via* T-type voltage-gated calcium channels (see Section above for full description) producing an inhibitory effect. A high DC (i.e., over 20%) during the on-periods will preferentially activate regular spiking (RS) pyramidal cells and fast spiking (FS) interneurons while suppressing the LTS interneurons producing an overall excitatory effect. This excitatory effect is simulated using a basic network model of LTS, FS, and RS neurons connected with excitatory and inhibitory synapses and thalamic inputs. The network model predicts an optimum excitation of 70% DC.

Neurons have a low elastic modulus (i.e., lower rigidity) (Elkin et al., 2007) and contain intracellular fluids, qualifying them as viscoelastic material with the ability to propagate mechanical energy *via* viscous dissipation of heat (i.e., the transformation of kinetic energy into internal energy) and store the mechanical energy elastically as the cells are deformed. These elastic properties allow for mechanical interactions at the cellular and subcellular levels including: intracellular and extracellular structures, cell cytoskeleton, extra-cellular matrix, and cell adhesion transmembrane proteins (Jerusalem et al., 2019). There are two mechanisms linked to the electrophysiological-mechanical coupling (for a comprehensive review see Jerusalem et al., 2019) in neuronal membrane: membrane conformational states (see Figure 2A) and mechanosensitive ion channels (see Figure 2D). Membrane conformational states are mechanical signals within the membrane configured by membrane tension, membrane elasticity, and viscosity of intracellular fluids. The membrane comformational states have three general frameworks for mechanisms of action: (1) voltage-induced changes due to membrane tension (see Figure 2A), (2) direct flexoelectricity (see Figure 2C), and (3) thermodynamic waves (see Figure 2B).

#### 3.1.1. Membrane Conformational States

There has been evidence (Tasaki and Iwasa, 1980, 1982; Tasaki et al., 1989; Mosbacher et al., 1998; Zhang et al., 2001; Kim et al., 2007; Gonzalez-Perez et al., 2016) for membrane conformational changes during action potentials. For example, axons swell during action potential generation and propagation (Kim et al., 2007).

Membrane displacements are possibly voltage-induced due to pressure differences between intracellular and extracellular fluids inducing changes in membrane curvature (Zhang et al., 2001; Mueller and Tyler, 2014) (Figure 2A, column 2). This change in curvature is hypothesized to have chemical (natural surface tension of the membrane) and electrical (stored energy in the membrane from its capacitance) components. These components are part of the phenomenon of electrowetting. Typically, when electrowetting occurs the surface tension of a liquid is altered (e.g., a spherical drop of water flattens due to the reduction of surface tension) when an electrical potential is applied, in neurons, a change in membrane potential modulates the surface tension of the membrane due to contact with intracellular and extracellular fluids. To maintain constant pressure across the membrane, membrane curvature must change due to the interface contact with intracellular and extracellular fluids (for a quantitative model see Mueller and Tyler, 2014).

These conformational changes can be exogenously induced *via* US's mechanical energy which induces mechanical phospholipid reconfigurations that ultimately changes the fluidity and permeability of the membrane (Taylor et al., 2017). These changes in the membrane's fluidity and permeability result in a high energy state causing embedded proteins and membrane lipids to adapt, altering conformational states and changing the capacitance of the membrane leading to a modulation of neural activity (Figure 2A, column 3).

A second mechanism of action for the electrophysiological-mechanical coupling is direct flexoelectricity (DF). DF is a property of dielectric materials allowing for the spontaneous electrical polarization from a mechanical strain (Zubko et al., 2013; Krichen and Sharma, 2016), similar to the piezoelectic effect. The electrical polarization occurs when the mechanical strain breaks the symmetry around the center of two-dimensional soft materials like a membrane (Krichen and Sharma, 2016). In biological DF (Nguyen et al., 2013; Deng et al., 2014; Ahmadpoor and Sharma, 2015), the lipid bilayers and cell membranes have phosopholipid molecules arranged into two sheets, which create dipole moments on the surface. Mechanical deformations to these surfaces cause a redistribution of dipoles and surface polarization (i.e., biological DF). In neurons, DF has been proposed as part of action potential propagation (Petrov, 1975; Petrov and Mircevova, 1986) (Figure 2C, column 2). US provides a possible membrane deformation, as demonstrated in cultured cells (Muratore et al., 2009) and artificial bilayers (Prieto et al., 2013), which could lead to DF (Figure 2C, column 3). Chen et al. (2019) demonstrated with computational modeling that high frequency oscillations can induce action potentials *via* DF.

The final potential mechanism of action for the electrophysiological-mechanical coupling is *via* a thermodynamic wave (Heimburg and Jackson, 2005, 2007). This thermodynamic wave (i.e., a soliton) is a mechanical pulse that propagates at a constant velocity and maintains its shape (Contreras et al., 2013) with two necessary conditions: speed varies as a function of frequency and as a non-linear function of pulse amplitude (Sassaroli and Vykhodtseva, 2016). These conditions are met due to the phase transition of the lipids in the cell membranes changing from a solid phase to liquid phase due to the melting point being just below the body temperature (Heimburg and Jackson, 2005, 2007). The lipids undergo a phase transition due to changes in ethalpy, entropy, volume, area, and thickness. The volume change is due to axonal swelling during an action potential enables the phase transition. This soliton model characterizes action potential propagation as an adiabatic process (i.e., heat does not enter nor leave the system); the energy at the source of the excitation is propagated adiabatically through the plasma membrane, resulting in the absence of net heat (i.e., total of all heat transfers in and out of the membrane) release from the initial temperature pulse during an action potential (Figure 2B, column 2). Exogenous US pulsation could interfere with these mechanical pulses by transferring it's acoustic energy from one molecule to the next, resulting in a pressure wave (Jerusalem et al., 2019) (Figure 2B, column 3). These pressure waves could generate action potentials or inhibit them depending the initial state of the neuron and its orientation in relation to the wave (El Hady and Machta, 2015).

### 3.2. Mechanosensitive Ion Channels

Mechanosensation is defined as the transduction of mechanical energy into neural signals *via* specialized sensory cells that can detect pressure with mechanosensitive ion channels like Transient receptor potential channels (TRP), which are activated by membrane stretching (Venkatachalam and Montell, 2007) (Figure 2D, column 2). US mechanical waves have been proposed to stretch these mechanosensitive ion channels due to the physical displacement from the mechanical waves (Mihran et al., 1990) causing reversible changes in ion transport mechanism and possibly depolarization (Tyler et al., 2008) (Figure 2D, column 3). Additionally, mechanosensitive ion channels from the two-pore-domain potassium channel family (e.g., TREK-1 and TRAAK channels) (Kubanek et al., 2016), along with ion channels that are not typically mechanosensitive (e.g., sodium and calcium voltage-gated channels, Morris and Juranka, 2007) have been shown to be responsive to US.

In mammals, there are a family of proteins in ion channel the *Homo sapiens* transient receptor potential A1 (*hs*TRPA1), which recently are found to have mechanosensitive properties (Duque et al., 2022). Duque and colleagues demonstrated that *in vitro* and *in vivo* sonication in rats and mice produced calcium influx and membrane currents in *hs*TRPA1-expressing mammalian human embryonic kidney-293T cells. A proposed mechanism of action for these sonication induced changes stems from the sonication sensitive N-terminal tip domain of the *hs*TRPA1 interaction actin cytoskeleton inducing changes in intracelluar calcium.

### 3.3. Microtubule Resonance

Hameroff et al. (2013) propose that US in specific megahertz frequency bands are within the resonance frequencies of microtubules, which allows for a vibration of said microtubules when the angle of approach aligns with their long-axis (Figure 2G). Given that microtubules are connected to actin filaments in dendritic spines (Lasser et al., 2018), US induced microtubules vibration could stand to modulate electrical signals by influencing synaptic plasticity.

### 3.4. Thermal Mechanism

HIFU's thermal mechanisms (Figure 2E) are well-understood given the technology's long standing use in ablation (Haar, 2010) related to the I_*spta*_ and steady-state temperature increases in the neuronal tissue (thermal index; TI) and in cranial bone (TIC) (Pasquinelli et al., 2019). In contrast, LIFU (Tables 1–**4**) would only produce fractional thermal increases ranging from 0.002 to 0.3 C (Yoo et al., 2011a; Lee et al., 2016b; Constans et al., 2018), unlikely capable of direct neuromodulation (Tyler et al., 2008; Wahab et al., 2012; Plaksin et al., 2014). However, a recent report (Darrow et al., 2019) shows a 2°C that may be a mechanism of action for neuroinhibition. Despite the unlikely nature of thermal mechanisms for neuromodulation, thermal modeling (Constans et al., 2018) can and should be employed to account for the different sonication parameters, tissue properties (e.g., density, perfusion, absorption coefficients), and beam/scanning configurations (Dalecki, 2004).

**Table 1:** Excitatory electrophysiology/neuroimaging findings for animals and humans.

**References**	**Subjects/target**	**Parameters**	**Major findings**
Tufail et al. (2010)	Mice (*n* = 11)	*f*_c_: 0.25 –0.5 MHz;	(1) EMG failure probability
	Motor cortex	I_SPPA_: 0.075–0.229 W/cm^2^;	increased with shorter ISI
		I_SPTA_: 0.021–0.163 W/cm^2^;	
		PRF: 1.2–3 kHz;	
		DC: 19–86%;	
		SD: 26–333 ms	
Yoo et al. (2011a)	Rabbits (*n* = 19)	*f*_c_: 0.69 MHz;	(1) Increased BOLD activity in
	Motor cortex	I_SPPA_: 3.3, 6.4, 9.5, 12.6 W/cm^2^;	Motor cortex using an
		I_SPTA_: 1.6, 3.2, 4.7, 6.3 W/cm^2^;	I_SPPA_ = 3.3 W/cm^2^
		PRF: 0.01 kHz;	
		DC: 50%;	
		SD: 500, 1,000, 1,500, 2,000 ms	
Kim et al. (2013)	Rats (*n* = 17)	*f*_c_: 0.35 MHz;	(1) Increase in glucose at
	Unilateral	I_SPPA_: 6 W/cm^2^;	sonication focal point
	Hemisphere	I_SPPA_: 3 W/cm^2^;	
		PRF: 1 kHz;	
		DC: 50%;	
		SD: 300 ms	
Kim et al. (2014a)	Rats (*n* = 7)	*f*_c_: 0.35 MHz;	(1) Increase in glucose was
	Motor cortex	I_SPPA_: 3 W/cm^2^;	smaller than the sonication
		I_SPTA_: 1.5 W/cm^2^;	focal point;
		PRF: 1 kHz;	(2) The average delay in tail
		DC: 50%;	movement was 171 (±63) ms
		SD: 300 ms	during sonication onset
Kim et al. (2014b)	Rats (*n* = 24)	*f*_c_: 0.35 MHz;	(1) Increase in magnitude of
	Visual area	I_SPPA_: 1, 3, and 5 W/cm^2^;	VEP at I_SPPA_ of 3 W/cm^2^ and
		I_SPTA_: 0.5, 1.5, and 2.5 W/cm^2^;	50% DC
		PRF: 0.1 kHz;	
		DC: 50%;	
		SE: 150s	
Lee et al. (2015b)	Sheep (*n* = 8)	*f*_c_: 0.25 MHz;	(1) Recorded MEP in hind leg
	Sensorimotor	I_SPPA_: 1.4–15.5 W/cm^2^;	muscle contralateral to
	Cortex	I_SPTA_: 0.7–7.75 W/cm^2^;	sonicated hemisphere with an
		PRF: 0.5 kHz;	I_SPPA_ of 6.9 W/cm^2^
		DC: 50%;	
		SD: 50–150 ms	
Lee et al. (2016c)	Sheep (*n* = 8)	*f*_c_: 0.25 MHz;	(1) Heterogeneity in MEP and
	Sensorimotor	I_SPPA_: 1.4–14.3 W/cm^2^;	VEP onset for each sheep with
	Cortex	I_SPTA_: 0.7–7.15 W/cm^2^;	an I_SPPA_ between 2–12 W/cm^2^;
		PRF: 0.5 kHz;	(2) Each sheep had increasing
		DC: 50%;	MEP and VEP intensities and
		SD: 300 ms	magnitudes when I_SPPA_ increased
Li et al. (2019)	Mice (*n* = 17)	*f*_c_: 2 MHz;	(1) Sonication induced action
	Primary	I_SPPA_: 46 W/cm^2^;	potentials at sonication location
	somatosensory	I_SPTA_: 0.7 W/cm^2^;	
	cortex	PRF: 1 kHz;	
		DC: 30%;	
		SD: 300 ms	
Yang et al. (2018)	Macaque (*n* = 2)	*f*_c_: 0.25 MHz;	(1) Similar BOLD activity
	Somatosensory	I_SPPA_: 9.9 W/cm^2^;	patterns for FUS and tactile
	cortex	I_SPTA_: 0.42 W/cm^2^;	stimulation;
		PRF: 2 kHz;	(2) FUS activated different
		DC: 50%;	network patterns than tactile
		SD: 3,000 ms (10 sonications)	stimulation
Sharabi et al. (2019)	Rats: Hamaline	*f*_c_: 0.23 MHz;	(1) Sonication induced motor
	induced (*n* = 5)	I_SPPA_: 27.2 W/cm^2^;	response in both normal and
	Sham (*n* = 8);	I_SPTA_: 0.816 W/cm^2^;	hamaline induced rats
	Rats (*n* = 5)	PRF: 0.03 kHz;	
	Oblongata	DC: 3%;	
	Medulla	SD: 100 ms	
Yoon et al. (2019)	Sheep (*n* = 10)	*f*_c_: 0.25 MHz;	(1) EMG response rates were
	Motor cortex	I_SPPA_: 15.8 and 18.2 W/cm^2^;	higher within contralateral leg
	Thalamus	I_SPTA_: 4.7, 5.5, 7.9, 9.1, 11.1,	vs. the ipsilateral leg;
		12.7, 15.8, 18.2 W/cm^2^;	(2) The 70% DC resulted in
		PRF: 0.1, 0.15, 0.16, 0.23, 0.25,	highest SEP and MEP response
		0.3, 0.5, 0.6, 0.7, 1, 1.4 kHz;	rates from US in motor cortex
		DC: 30, 50, 70, 100%;	and thalamus;
		SD: 60, 100, 140, 200 ms	(3) There was no significant difference between intensities; however, I_SPPA_ of 15.8W/cm^2^ generated more responses for MEPs than the I_SPPA_ of 18.2W/cm^2^;
			(4) The 1.4 kHz PRF resulted in highest response rate in SEPs and MEPs for US of motor cortex and thalamus
Yu et al. (2021)	Rats (*n* = 9)	*f*_c_: 0.5 MHz;	(1) Excitatory neurons
	Somatosenory	I_SPPA_: 50 W/cm^2^;	increased spike rates with
	cortex	I_SPTA_: 3, 15, 30, 45 mW/cm^2^;	higher PRFs and DCs
		PRF: 0.03, 0.3, 1.5, 3, 4.5 kHz;	
		DC: 0.6, 6, 30, 60, 90%;	
		SE: 67 ms	
Lee et al. (2016b)	Human (*n* = 19)	*f*_c_: 0.27 MHz;	(1) Increased BOLD activation
	Visual Cortex	I_SPPA_: 0.7–6.6 W/cm^2^;	in V1 during sonication;
		I_SPTA_: 0.35–3.3 W/cm^2^;	(2) Sonication evoked EEG
		PRF: 0.5 kHz;	potentials similar to VEP;
		DC: 50%;	(3) Sensory perception of
		SD: 300 ms	phosphenes
Ai et al. (2016)	Human (*n* = 6)	*f*_c_: 0.5 MHz;	(1) Increased BOLD activation
	Sensorimotor	I_SPPA_: 6 W/cm^2^;	in sensorimotor regions
	cortex	I_SPTA_: 2.16 W/cm^2^;	
		PRF: 1 kHz;	
		DC: 36%;	
		SD: 500 ms	
Ai et al. (2016)	Human (*n* = 6)	*f*_c_: 0.86 MHz;	(1) Increased BOLD activation
	Caudate	I_SPPA_: 6 W/cm^2^;	in caudate
		I_SPTA_: 3 W/cm^2^;	
		PRF: 0.5 kHz;	
		DC: 50%;	
		SD: 500 ms	
Ai et al. (2018)	Human (*n* = 5)	*f*_c_: 0.5 MHz;	(1) Increased BOLD activation
	Motor cortex	I_SPPA_: 16.95 W/cm^2^;	in motor cortex's finger
		I_SPPA_: 6.102 W/cm^2^;	representation;
		PRF: 1 kHz;	(2) Activity did not spread to
		DC: 36%;	functionally connected motor
		SD: 500 ms	regions
Gibson et al. (2018)	Human (*n* = 19)	*f*_c_: 2.32 MHz;	(1) Increased cortical
	Motor cortex	I_SPPA_: 34.96 W/cm^2^;	excitability of M1 following
	Sham (*n* = 21)	I_SPTA_: 132.85 mW/cm^2^;	sonication that lasted 360 s;
		DC: 100%;	(2) Cortical excitability did not
		SE: 2 min	increase 660 s post-sonication
Lee et al. (2015a)	Human (*n* = 18)	*f*_c_: 0.25 MHz;	(1) Sonication induced cortical
	Somatosensory	I_SPPA_: 3 W/cm^2^;	evoked potentials similar to
	cortex	I_SPTA_: 1.5 W/cm^2^;	SEP response from medial
		PRF: 0.5 kHz;	nerve stimulation
		DC: 50%;	
		SD: 300 ms	
Liu et al. (2021)	Humans (*n* = 9)	*f*_c_: 0.5 MHz;	(1) Increased amplitude of
	Somatosenosry	I_SPPA_: 5.64 W/cm^2^;	N300 component source
	cortex	I_SPTA_: 0.338 W/cm^2^;	localized in the somatosensory
		PRF: 0.3 kHz;	cortex
		DC: 6%	
Yuan et al. (2020)	Mice (*n* = 29)	*f*_c_: 0.5 MHz;	(1) Peak CBF monotonically
	Motor cortex	I_SPPA_: 0.2, 0.4, 0.8, 1.1 W/cm^2^;	increased with I_SPPA_ or SD;
		I_SPTA_: 0.08–0.44 W/cm^2^;	
		PRF: 1 kHz	
		DC: 10, 20, 30, 40%;	
		SD: 50, 100, 200, 300, 400 ms	
Yang et al. (2021)	Macaque (*n* = 2)	*f*_c_: 0.25 MHz;	(1) Sonication induced BOLD
	Somatosensory	I_SPPA_: 6 W/cm^2^;	activation increase in primary
	cortex	I_SPTA_: 0.0271 W/cm^2^;	and secondary somatosensory,
		PRF: 2 kHz;	posterior insular, and
		DC: 50%;	midcingulate cortices during rest
Lu et al. (2020)	Rats (*n* = 6)	*f*_c_: 0.5 MHz;	(1) Sonication induced low
	Visual cortex	I_SPPA_: 115.8 W/cm^2^;	frequency activations with
	Retinal	I_SPTA_: 28.9, 38.6, 57.9 mW/cm^2^;	four peaks (N1, P1, N2, P2)
	degenerate	PRF: 0.1, 0.2, 0.333, 0.5 kHz;	except with PRF of 0.1 kHz;
	rats (*n* = 11)	DC: 25, 33.3, 40, 50%;	(2) Retinal degenerate rats
		SE: 67 ms	had larger recorded amplitudes of visual cortex neurons than the control rats during sonication

### 3.5. Cavitation and Sonoporation

An ultrasonic stimulation with sufficient intensity (related to I_*sppa*_) resonates, expands, and collapses gas bubbles within tissues causing cavitation (Dalecki, 2004; Krasovitski et al., 2011) (Figure 2F). Non-inertial/stable cavitation is a mechanical effect that creates a stable oscillation of gas bubbles at multiple frequencies. Inertial cavitation is a sudden collapse from rapid expansion due to high exposure amplitudes generating decompression (rarefaction pressure) from the interaction of the acoustic pressure wave with the tissue (Dalecki, 2004) measured with the mechanical index (MI; a proportion of the peak negative pressure over square root of the characteristic frequency, which means lower frequencies have a higher MI). These effects are unlikely in the nervous system due to the general lack of gas bubbles. Microcavitation could still occur in neurons, leading to sonoporation (Figure 2F), by increasing membrane permeability *via* the creation of pores in the lipid bilayer.

Based on microcavitation, there are two models for US modulation: intramembrane cavitation hypothesis (Krasovitski et al., 2011) and neuronal intramembrane cavitation excitation (NICE) model (Plaksin et al., 2014) (Figure 3). The intramembrane cavitation hypothesis describes bilayer sonophores (i.e., small intramembrane regions) that allow US oscillatory expansions to cause capacitive changes caused by the frequency and acoustic pressure of the US, which build up over a millisecond time scale leading to the neuron reaching its threshold, causing an action potential. Plaksin et al. (2016) expanded their NICE model (Figure 3) for multiple types of excitatory cortical neurons (i.e., regular spiking pyramidal), inhibitory neurons (i.e., low threshold spiking and fast spiking) and thalamic neurons (i.e., thalamocortical and thalamic reticular). During US on-periods (Figure 3, High DC), regular spiking neurons' excitation is driven by US-induced membrane potential oscillations due to voltage-gated ion channels being closed but non-voltage gated channels remaining open and fluctuating leak currents increasing the membrane's charge (Plaksin et al., 2014). Following the cessation of US, the membrane returns to its reference capacitance, which allows for the membrane's charge to determine the membrane's potential leading to an action potential or multiple action potentials due to a longer duration. In the case of inhibition (Figure 3, Low DC), the low threshold spiking neurons have T-type voltage-gated calcium channels with voltage-gated channels containing fast gates (S-gates) and slower gates (U-gates) (Huguenard and McCormick, 1992). These temporal differences allow for boosting charge accumulation during the off periods between short US bursts. Thus, DC determines excitation (higher DC, allowing for longer sonication-on periods) and inhibition (lower DC, allowing for short US bursts with longer periods between bursts) independent of the other sonication parameters. Finally, according to the network model (Figure 3), excitation occurs optimally at a DC of 70%, allowing for a trade-off in regular-spiking and fast-spiking neurons between charge accumulation during the US and discharge during the off periods of US.

## 4. Empirical Findings

### 4.1. Excitatory

Table 1 contains excitatory findings with sonication targets located in the caudate (Ai et al., 2016), medulla oblongata (Sharabi et al., 2019), motor cortex (Tufail et al., 2010; Yoo et al., 2011a; Kim et al., 2013; Ai et al., 2018; Gibson et al., 2018; Yoon et al., 2019; Yuan et al., 2020), sensorimotor regions (Lee et al., 2015b, 2016c; Ai et al., 2016), somatosensory cortex (Lee et al., 2015a; Yang et al., 2018, 2021; Li et al., 2019; Yu et al., 2021; Liu et al., 2021), thalamus (Yoon et al., 2019), and visual cortex (Kim et al., 2015; Lee et al., 2016b; Lu et al., 2020). Yoon et al. (2019) found that 70% DC produced the highest response rates of motor evoked potentials (MEPs) and sensory evoked potentials (SEPs), while there are other alterations to intensity, PRF, and SD on response rate of MEPs or SEPs without a high DC. Yu et al. (2021) found that high PRF and high DC resulted in higher spike rates recorded with implanted neural recording arrays. Unfortunately, PRF and DC both monotonically increased, so the effects of each are not dissociable. Both of these studies validate the proposed dependence of excitation on higher DCs predicted by the modified NICE model (Plaksin et al., 2016). However, Yoon et al. (2019) found that a DC of 100% decreased response rate, which was not predicted by the modified NICE model. Furthermore, over half the studies with DC above 10% (Ai et al., 2018; Gibson et al., 2018; Yang et al., 2018, 2021; Li et al., 2019; Sharabi et al., 2019; Yoon et al., 2019; Yu et al., 2021; Lu et al., 2020; Liu et al., 2021) were published after the modification of the NICE model.

### 4.2. Suppressive

Table 2 highlights the studies reviewed for their suppressive findings that had sonication targets in the auditory cortex (Daniels et al., 2018), inferior colliculus (Daniels et al., 2018), globus pallidus (Cain et al., 2021), the motor cortex (Yoo et al., 2011a; Legon et al., 2018b; Yoon et al., 2019), the somatosenory cortex (Legon et al., 2014; Chu et al., 2015; Yu et al., 2021; Yang et al., 2021), the thalamus (Legon et al., 2018a; Darrow et al., 2019; Yoon et al., 2019), and visual areas (Kim et al., 2015). Yoon et al. (2019) findings using suppressive sequences at low DC while PRF varied support the modified NICE model's predictions for low DC preferentially targeting low threshold spiking neurons (Plaksin et al., 2016). However, in another effort, Yu et al. (2021) found that inhibitory neurons in the somatosensory cortex had high spike rates regardless of the PRF or DC. These findings, along with the observation of suppressive effects on (a) short-latency and late-onset SEP responses (Legon et al., 2014), (b) MEP amplitudes (Legon et al., 2018b), (c) reduced somatosensory evoked potential (SSEP) amplitudes (Darrow et al., 2019) and (d) the P14 component of SEP (Legon et al., 2018a), using a DC in the excitatory range, do not support the modified NICE model's for inhibition. Overall, other than findings published before Plaksin et al. (2016), the modified NICE model is only supported by Yoon et al. (2019) study of parameter space and by Daniels et al. (2018) research on the suppression of auditory evoked potentials (AEP) because they evoked suppressive effects using a low DC preferentially activating inhibitory neurons.

**Table 2:** Suppressive findings for animals and humans.

**References**	**Subjects/target**	**Parameters**	**Major findings**
Yoo et al. (2011a)	Rabbits (*n* = 19)	*f*_c_: 0.69 MHz;	(1) Sonication induced
	Motor cortex	I_SPPA_: 3.3 and 6.4 W/cm^2^;	reduction in VEP magnitude
		I_SPTA_: 0.165 and 0.32 W/cm^2^;	for the P30 component
		PRF: 0.1 kHz;	
		DC: 5%;	
		SD: 7,000–8,000 ms	
Chu et al. (2015)	Rats (*n* = 118)	*f*_c_: 0.4 MHz;	(1) Sonication induced
	Somatosensory	MI: 0.3, 0.55, 0.8;	reduction in SSEP magnitude
	cortex	PRF: 0.01 kHz;	(2) Sonication induced
		DC: 1%;	reduction in SSEP magnitude
		SD: 10 ms	for 60 min with a 0.55 MI
Kim et al. (2015)	Rats (*n* = 24)	*f*_c_: 0.35 MHz;	(1) Decrease in VEP magnitude
	Visual area	I_SPPA_: 1, 3, and 5 W/cm^2^;	at I_SPPA_ of 3 W/cm^2^ & 5% DC;
		I_SPTA_: 0.03–0.25 W/cm^2^;	(2) Lower DC and intensity
		PRF: 0.1 kHz;	combinations did not produce
		DC: 1, 5, 8.3%;	VEP suppression effects
		SE: 150s	
Legon et al. (2014)	Human (*n* = 10)	*f*_c_: 0.5 MHz;	(1) Sonication induced
	Somatosenory	I_SPPA_: 5.9 W/cm^2^;	modulation of short-latency
	cortex	I_SPTA_: 2.124 W/cm^2^;	and late-onset SEP responses
		PRF: 1 kHz;	
		DC: 36%;	
		SD: 500 ms	
Legon et al. (2018b)	Human (*n* = 50)	*f*_c_: 0.5 MHz;	(1) Sonication induced
	Motor cortex	I_SPPA_: 17.2 W/cm^2^; l	reduction of MEP and
		I_SPTA_: 6.192 W/cm^2^;	intracortical facilitation;
		PRF: 1 kHz;	(2) Sonication did not induce
		DC: 36%;	significant changes to short-
		SD: 500 ms	interval intracortical inhibition
Legon et al. (2018a)	Human (*n* = 40)	*f*_c_: 0.5 MHz;	(1) In SEP, sonication induced
	Thalamus	I_SPPA_: 7.02 W/cm^2^;	reduction of P14 component;
		I_SPTA_: 2.53 W/cm^2^;	(2) Sonication induced
		PRF: 1 kHz;	attenuation in alpha, beta, and
		DC: 36%;	gamma power bands
		SD: 500 ms	
Daniels et al. (2018)	Rats (*n* = 22);	*f*_c_: 0.23 MHz;	(1) In AEP, sonication induced
	Pigs (*n* = 5)	I_SPPA_: 2.3 and 4.6 W/cm^2^;	reduction in all animals;
	Inferior	I_SPTA_: 0.07 and 0.14 W/cm^2^;	(2) Suppression was weaker in
	colliculus	PRF: 1 kHz;	rats at the lower intensity
	Auditory cortex	DC: 3%;	
		SD: 100 ms	
Yoon et al. (2019)	Sheep (*n* = 10)	*f*_c_: 0.25 MHz;	(1) Reduction in SEP
	Motor cortex	I_SPPA_: 5.4 and 11.6 W/cm^2^;	magnitude of 18–35%
	Thalamus	I_SPTA_: 0.16, 0.35, 0.58 W/cm^2^;	using an I_SPPA_ of 5.4 W/cm^2^,
		PRF: 0.03, 0.05, 0.06, 0.1 kHz;	and a 3 or 5% DC and a PRF
		DC: 3 and 5 %;	of 0.06 or 0.1 kHz;
		SD: 200 ms	(2) SEP reduction lasted approximately 5 min
Yu et al. (2021)	Rats (*n* = 9)	*f*_c_: 0.5 MHz;	(1) Inhibitory neurons have
	Somatosenory	I_SPPA_: 50 mW/cm^2^;	high spike rates across all
	cortex	I_SPTA_: 3, 15, 30, 45 mW/cm^2^;	PRFs and DCs
		PRF: 0.03, 0.3, 1.5, 3, 4.5 kHz;	
		DC: 0.6, 6, 30, 60, 90 %;	
		SD: 67 ms	
Darrow et al. (2019)	Rats (*n* = 1)	*f*_c_: 3.2 MHz;	(1) Sonication induced SSEP
	Thalamus	I_SPTA_: 0.01–88 W/cm^2^;	suppression increases with
		PRF: 0.5 kHz;	intensity, but unrelated to DC;
		DC: 5–70%	(2) Thermal changes of up 2°C during sonication induced suppression of SSEP
Fomenko et al. (2020)	Human (*n* = 18)	*f*_c_: 0.5 MHz;	(1) Sonication induced MEP
	Motor cortex	I_SPPA_: 2.32 W/cm^2^;	suppression using DC of 10%
		I_SPTA_: 0.23, 0.69, 1.16 W/cm^2^;	with a SD of 0.4 and 0.5 s only
		PRF: 0.2, 0.5, 1 kHz;	
		DC: 10, 30, 50%	
		SD: 0.1, 0.2, 0.3, 0.4, 0.5 s	
Cain et al. (2021)	Human (*n* = 16)	*f*_c_: 0.65 MHz;	(1) During sonication, the
	Left Globus	I_SPPA_: 14.4 W/cm^2^;	left globus pallidus had reduced
	Pallidus	I_SPTA_: 0.72 W/cm^2^;	BOLD using 0.1 kHz PRF;
		PRF: 0.1 and 0.01 kHz;	(2) Relative perfusion in left
		DC: 5%;	globus pallidus was decreased
		SE: 30 s per sonication	post-sonication uisng 0.1 kHz
		(10 total sonications)	PRF
Yang et al. (2021)	Macaque (*n* = 2)	*f*_c_: 0.25 MHz;	(1) Sonication induced reduced
	Somatosensory	I_SPPA_: 6 W/cm^2^;	BOLD activations of primary
	cortex	I_SPTA_: 0.0271 W/cm^2^;	and secondary somatosensory,
		PRF: 2 kHz;	posterior insular, and
		DC: 50%;	midcingulate cortices during peripheral tactile stimulation

### 4.3. Behavioral

Table 3 presents the studies with behavioral findings of decreased time to voluntary movement following anesthesia following thalamic stimulation (Yoo et al., 2011b), changes in performance on discrimination tasks during stimulation of frontal eye fields (FEF) and somatosensory cortex (Legon et al., 2014; Kubanek et al., 2020; Liu et al., 2021), limb movements following stimulation of motor cortex (Tufail et al., 2010; Yoo et al., 2011a; Li et al., 2019; Yuan et al., 2020), stimulus response reduced reaction time during stimulation of motor cortex (Fomenko et al., 2020), and increased language comprehension for a patient with a disorder of consciousness following stimulation thalamus (Monti et al., 2016). When these studies are grouped as a function of DC, in accordance with the modified NICE model (Plaksin et al., 2016), a majority of the studies have DC values high enough to produce excitatory neuromodulation, yielding the following behavioral findings: limb movements (Tufail et al., 2010; Yoo et al., 2011a; Li et al., 2019), and right bias toward leftward choices (Kubanek et al., 2020). The observations that (a) excitation of neurons in the primary motor cortex are needed to induce limb movements, and (b) the right bias toward leftward choices have the opposite polarity of previous findings using neuroinhibitive drugs (Schiller and Tehovnik, 2003; Kubanek et al., 2015) suggests that the neurmodulatory effects were excitatory.

**Table 3:** Behavioral findings for animals and humans.

**References**	**Subjects/target**	**Parameters**	**Major findings**
Kim et al. (2014a)	Rats (*n* = 37)	*f*_c_: 0.35 and 0.65 MHz;	(1) Sonication induced tail
	Motor cortex	I_SPPA_: 4.9–22.4 W/cm^2^;	movement using a DC of 50%
		I_SPTA_: 1–11.2 W/cm^2^;	with an I_SPPA_ between 4.9 and
		PRF: 0.06–2.8 kHz;	5.6 W/cm^2^
		DC: 30–100%;	
		SD: 150–400 ms	
Yoo et al. (2011b)	Rats (*n* = 19)	*f*_c_: 0.65 MHz;	(1) Decreased time to voluntary
	Thalamus	I_SPPA_: 3.3 and 6 W/cm^2^;	movement and pinch response
		I_SPTA_: 0.17 and 0.3 W/cm^2^;	with an I_SPPA_ = 3.3 W/cm^2^;
		PRF: 0.1 kHz;	(2) Decreased anesthetic
		DC: 5%	duration with an I_SPPA_ of 6 W/cm^2^
Kubanek et al. (2020)	Macaque (*n* = 2)	*f*_c_: 0.27 MHz;	(1) Sonication induced bias
	FEF	I_SPPA_: 11.6 W/cm^2^;	toward rightward and leftward
		I_SPTA_: 0.581 W/cm^2^;	choices congruent to the
		PRF: 0.5 kHz;	stimulation laterality indicating
		DC: 50%;	possible neuronal excitation
Li et al. (2019)	Mice (*n* = 17)	*f*_c_: 2 MHz;	(1) Head-turning behavior
	Somatosensory	I_SPPA_: 46 W/cm^2^;	during sonication
	cortex	I_SPTA_: 13.8 W/cm^2^;	
		PRF: 1 kHz;	
		DC: 30%;	
		SD: 300 ms	
Tufail et al. (2010)	Mice (*n* = 11)	*f*_c_: 0.25–0.5 MHz;	(1) Limb Movements;
	Motor cortex	I_SPPA_: 0.075–0.229 W/cm^2^;	(2) No significant changes in
		I_SPTA_: 0.021–0.163 W/cm^2^;	wire-hanging or rotorod task
		PRF: 1.2–3 kHz;	performance
		DC: 19–86%;	
		SD: 26–333 ms	
Yoo et al. (2011a)	Rabbits (*n* = 19)	*f*_c_: 0.69 MHz;	(1) Limb movement using an
	Motor cortex	I_SPPA_: 3.3, 6.4, 9.5, 12.6 W/cm^2^;	I_SPPA_ of 12.6 W/cm^2^
		I_SPTA_: 1.65, 3.2, 4.75, 6.3 W/cm^2^;	
		PRF: 0.01 kHz;	
		DC: 50%;	
		SD: 500, 1,000, 1,500, 2,000 ms	
Legon et al. (2014)	Human (*n* = 10)	*f*_c_: 0.5 MHz;	(1) Increased performance on
	Somatosenory	I_SPPA_: 5.9 W/cm^2^;	discrimination task without
	Cortex	I_SPTA_: 2.12 W/cm^2^;	affecting attention or response
		PRF: 1 kHz;	bias
		DC: 36%;	
		SD: 500 ms	
Legon et al. (2018a)	Human (*n* = 50)	*f*_c_: 0.5 MHz;	(1) Sonication induced
	Motor cortex	I_SPPA_: 17.2 W/cm^2^;	reduction of reaction time
		I_SPTA_: 6.19 W/cm^2^;	
		PRF: 1 kHz;	
		DC: 36%;	
		SD: 500 ms	
Legon et al. (2018b)	Human (*n* = 40)	*f*_c_: 0.5 MHz;	(1) Sonication induced
	Thalamus	I_SPPA_: 7.02 W/cm^2^;	reduction of discrimination and
		I_SPTA_: 2.53 W/cm^2^;	performance on two-point
		PRF: 1 kHz;	discrimination task
		DC: 36%;	
		SD: 500 ms	
Monti et al. (2016)	Human (*n* = 1)	*f*_c_: 0.65 MHz;	(1) In 3 days post-sonication,
	Thalamus	I_SPPA_: 14.4 W/cm^2^;	the patient displayed increased
		I_SPTA_: 0.72 W/cm^2^;	language comprehension with
		PRF: 0.1 kHz;	reliable responses to commands
		DC: 5%;	and ability to communicate;
		SE: 30 s per sonication	(2) In 11 days post sonication,
		(10 total sonications)	the patient attempted to walk
Fomenko et al. (2020)	Human (*n* = 18)	*f*_c_: 0.5 MHz;	(1) Reduction in reaction
	Motor cortex	I_SPPA_: 2.32 W/cm^2^;	time in visual task
		I_SPTA_: 0.23, 0.69, 1.16 W/cm^2^;	
		PRF: 0.2, 0.5, 1 kHz;	
		DC: 10, 30, 50%	
		SD: 0.1, 0.2, 0.3, 0.4, 0.5s	
Liu et al. (2021)	Humans (*n* = 9)	*f*_c_: 0.5 MHz;	(1) Increased accuracy of
	Somatosenosry	I_SPPA_: 5.64 W/cm^2^;	vibration frequency
	cortex	I_SPTA_: 0.33828 W/cm^2^;	discrimination
		PRF: 0.3 kHz;	
		DC: 6%	
Yuan et al. (2020)	Mice (*n* = 29)	*f*_c_: 0.5 MHz;	(1) Whisker and tail movement
	Motor cortex	I_SPPA_: 0.2, 0.4, 0.8, 1.1 W/cm^2^;	during and after sonication
		I_SPTA_: 0.08–0.44 W/cm^2^;	with any parameter set
		PRF: 1 kHz;	
		DC: 10, 20, 30, 40%;	
		SD: 50, 100, 200, 300, 400 ms	

### 4.4. Other

Table 4 showcases US effects ranging from mood alterations (Hameroff et al., 2013; Sanguinetti et al., 2020), to pain reduction (Hameroff et al., 2013), lesion reduction (Guo et al., 2015), reduction of systolic blood pressure (Li et al., 2020), reduction of anhedonia (Zhang et al., 2018), inducing tactile sensation (Lee et al., 2016a), enhanced cortical-muscular coupling (Xie et al., 2018), producing long-lasting effects (up to 35 min) in SEP responses (Yoo et al., 2011a) or reduction (up to 2 days at highest intensities) of fMRI BOLD responses (Chu et al., 2015), excitation and inhibition with the same sonication pulse (Wattiez et al., 2017), modulation of power bands using local field potentials (LFP) recorded with electroencephalogram (EEG) (Legon et al., 2014; Wang et al., 2019), and functional connectivity changes between sonication targets and other cortical regions (Folloni et al., 2019; Verhagen et al., 2019; Sanguinetti et al., 2020). There exist a host of potential mechanisms for ultrasound-induced neuromodulation underlying the breadth of electrophysiological, behavioral, cognitive, mood, and connectivity findings discussed herein, but the modified NICE model (Plaksin et al., 2016) allows for a grouping parameter (DC) at which effects can be assessed categorically. The studies reporting a DC in the range of inhibitory stimulation found long-lasting effects on BOLD and SEP responses (Yoo et al., 2011a; Chu et al., 2015), while the remaining reported DCs within the excitatory ranges. Wattiez et al. (2017) stimulated FEF in two macaques while recording single neurons in supplemental eye fields (SEF; a region directly connected to FEF). The sonication induced excitation in approximately half the neurons recorded in SEF and inhibition in the other half.

**Table 4:** Other findings for animals and humans.

**References**	**Subjects/target**	**Parameters**	**Major findings**
Wattiez et al. (2017)	Macaque (*n* = 2)	*f*_c_: 0.32 MHz;	(1) Increased nerual activity in
	FEF	I_SPPA_: 1.9 and 5.6 W/cm^2^;	47% and 53% of recorded
		I_SPTA_: 1.9 and 5.6 W/cm^2^;	SEF neurons
		DC: 100%;	(2) The remaining recorded
		SD: 100 ms	SEF neurons decreased in activity for each macaque monkey
Guo et al. (2018)	Guinea pigs	*f*_c_: 0.22 MHz;	(1) Sonication induced
	(*n* = 2)	I_SPPA_: 0.02–0.33 W/cm^2^;	activation of multiple cortical
	Somatosensory,	I_SPTA_: 0.00004–0.0198 W/cm^2^;	and sub-cortical regions;
	auditory, visual	PRF: 0.01–16 kHz;	(2) Elimination of US elicited
	cortices	DC: 0.2–60%;	cortical and sub-cortical
		SD: 500 ms	activity after removal of cochlear fluids or transection of auditory nerve
Sato et al. (2018)	Mice (*n* = 20)	*f*_c_: 0.5 MHz;	(1) Sonication induced
	Somatosensory	I_SPPA_: 14 W/cm^2^;	activation of multiple cortical
	auditory, visual	I_SPTA_: 0.03, 0.11, 0.38, 1.3, 4.2	and sub-cortical regions;
	cortices	W/cm^2^;	(2) Elimination of US elicited
		PRF: 1.5 kHz;	cortical and sub-cortical
		DC: 0.81, 2.7, 9, and 30%;	activity
		SD: 500 ms	
Chu et al. (2015)	Rats (*n* = 118)	*f*_c_: 0.4 MHz;	(1) Reduction in BOLD for
	Somatosensory	MI: 0.3, 0.55, 0.8;	2 days at intensity of 0.8 MI,
	cortex	PRF: 0.01 kHz;	and transient reduction of
		DC: 1%;	BOLD with a 0.55 MI;
		SD: 10 ms	(2) No reduction of BOLD with a 0.33 MI and for control group
Zhang et al. (2018)	Rats with	*f*_c_: 0.5 MHz;	(1) Sonication induced
	depression	I_SPPA_: 7.59 W/cm^2^;	reduction in anhedonia
	(*n* = 76)	I_SPTA_: 4.55 W/cm^2^;	and exploratory behavior
	Prefrontal	PRF: 1.5 kHz;	
	cortex	DC: 60%;	
Guo et al. (2015)	Ischemic rats	*f*_c_: 0.5 MHz;	(1) Reduction of ischemic
	(*n* = 38)	I_SPPA_: 0.44 W/cm^2^;	lesion following sonication;
	Ischemic core	I_SPTA_: 0.057 W/cm^2^;	(2) Reduction of cortical infarct
		DC: 13.33%;	volume compared to control
		PRF: 1.5 kHz;	group
		SD: 400 ms	
Xie et al. (2018)	Mice (*n* = 9)	*f*_c_: 0.5 MHz;	(1) Sonication induced
	Motor cortex	I_SPPA_: 1.1 W/cm^2^;	enhancing of cortico-muscular
		I_SPTA_: 0.275 W/cm^2^;	coupling with increasing
		PRF: 1 kHz;	number of tone bursts
		DC: 25%	
Yoo et al. (2011a)	Rats (*n* = 11)	*f*_c_: 0.65 MHz;	(1) Sonication induced SEP
	Somatosenosry	I_SPPA_: 4.2 W/cm^2^;	modulation lasting over 35 min
	cortex	I_SPTA_: 0.21 W/cm^2^;	
		PRF: 0.1 kHz;	
		DC: 5%;	
		SE: 30 min	
Wang et al. (2019)	Mice (*n* = 33)	*f*_c_: 0.5 MHz;	(1) A decrease in relative power
	Motor cortex	I_SPPA_: 0.2, 0.4, 0.8, 1.1 W/cm^2^;	in theta band as I_SPPA_ increases;
		I_SPTA_: 0.08, 0.11, 0.16, 0.2, 0.3,	(2) Relative power of both
		0.44 W/cm^2^;	gamma and high gamma bands
		PRF: 1 kHz;	increasing with I_SPPA_ increases
		DC: 10, 20, 30, 40%;	
		SD: 100, 200, 300, 400 ms	
Folloni et al. (2019)	Macaque (*n* = 9)	*f*_c_: 0.25 MHz;	(1) Sonication induced
	Anterior	I_SPPA_: 18.8 and 64.9 W/cm^2^;	reduction of functional
	cingulate cortex	I_SPTA_: 5.64 and 19.47 W/cm^2^;	coupling in amygdala and other
	and amygdala	PRF: 0.01 kHz;	cortical regions lasting an hour;
		DC: 30%	(2) Sonication induced reduction of functional connectivity between anterior cingulate cortex and other brain regions lasting an hour
Mohammadjavadi et al. (2019)	Deaf mice	*f*_c_: 0.500 MHz;	(1) Sonication induced EMG
	(*n* = 11);	I_SPPA_: 1, 2.79, 3.78 W/cm^2^;	response are the same for both
	Mice (*n* = 21)	I_SPTA_: 0.8, 2.23, 3.02 W/cm^2^;	deaf and normal mice;
	Motor cortex	PRF: 1.5 and 8 kHz;	(2) EMG motor response
	DC: 80%		duration was positively correlated with sonication exposure time;
			(3) US with rectangular envelope can activates peripheral auditory pathways, but smoothing the envelop eliminates this activation
Verhagen et al. (2019)	Macaque (*n* = 6)	*f*_c_: 0.25 MHz;	(1) Sonication induced one
	SMA and FPC	I_SPPA_: 24.1 and 31.7 W/cm^2^;	hour modulation of functional
		I_SPTA_: 7.23 and 9.51 W/cm^2^;	coupling between SMA and
		PRF: 0.01 kHz;	other cortical regions;
		DC: 30%	(2) Sonication induced modulation of functional connectivity between FPC and other brain regions lasting an hour;
			(3) Sonication induced activation of FPC and SMA
Hameroff et al. (2013)	Humans with	*f*_c_: 8 MHz;	(1) Sonication induced
	chronic pain	I_SPTA_: 0.15 W/cm^2^;	improved mood;
	(*n* = 14)	SE: 15s	(2) Reduction of pain after 40
	frontal cortex		min following sonication
	contralateral to maximal pain		
Legon et al. (2014)	Human (*n* = 10)	*f*_c_: 0.5 MHz;	(1) Sonication induced
	Somatosenory Cortex	I_SPPA_: 5.9 W/cm^2^;	modulation of late-onset alpha,
		I_SPTA_: 2.12 W/cm^2^;	beta, and gamma bands
		PRF: 1 kHz;	occurred 200 ms following
		DC: 36%;	sonication
		SD: 500 ms	
Lee et al. (2015a)	Human (*n* = 18)	*f*_c_: 0.25 MHz;	(1) Sonication did not
	Somatosensory	I_SPPA_: 3 W/cm^2^;	induce tactile sensations
	cortex	_SPTA_: 1.5 W/cm^2^;	
		PRF: 0.5 kHz;	
		DC: 50%;	
		SD: 300 ms	
Lee et al. (2016a)	Human (*n* = 10)	*f*_c_: 0.21 MHz;	(1) Sonication induced tactile
	Somatosensory	I_SPPA_: 7–8.8 W/cm^2^;	sensations
	cortices	I_SPTA_: 3.5–4.4 W/cm^2^;	
		PRF: 0.5 kHz;	
		DC: 50%;	
		SD: 500 ms	
Sanguinetti et al. (2020)	Human (*n* = 24)	*f*_c_: 0.5 MHz;	(1) Sonication induced positive
	Right inferior	I_SPPA_: 54 W/cm^2^;	mood reflected in VAMS;
	frontal gyrus	I_SPTA_: 0.13 W/cm^2^;	(2) Sonication induced
	Sham (*n* = 24)	PRF: 0.04 kHz;	reduction of functional
		DC:26%;	connectivity in mood and
		SD: 30s	emotion regulation resting state networks
Cain et al. (2021)	Human (*n* = 16)	*f*_c_: 0.65 MHz;	(1) During sonication, the
	Left Globus	I_SPPA_: 14.4 W/cm^2^;	primary somatosenosry cortex,
	Pallidus	I_SPTA_: 0.72 W/cm^2^;	cingulate cortex, and left
		PRF: 0.1 and 0.01 kHz;	thalamus had reduced BOLD
		DC: 5%;	using 0.1 kHz PRF;
		SE: 30 s per sonication	(2) Relative perfusion in
		(10 total sonications)	putamen and thalamus was decreased post-sonication uisng 0.1 kHz PRF
Li et al. (2020)	Hypertensive	*f*_c_: 0.62 MHz;	(1) Post-sonication increase of
	Rats (*n* = 32)	I_SPPA_: 5.13 W/cm^2^;	c-fos proteins in ventrolateral
	Ventrolateral	I_SPTA_: 2.56 W/cm^2^;	periaquiductal gray and caudal
	periaquiductal	PRF: 0.25 kHz;	ventrolateral medulla
	gray	DC: 50%;	(2) Decreased mean systolic blood pressure

Several studies have also explored the possibility of an auditory confound stemming from the plausibility that the high-frequency acoustic noise could stimulate auditory pathways. Guo et al. (2018) found that sonication-induced cortical and subcortical activations were eliminated when the animals had either their cochlear fluid removed or auditory nerve transected. Sato et al. (2018) also found that chemical deafening of mice eliminated sonication-induced cortical activations. However, Mohammadjavadi et al. (2019) were able to induce motor responses with US in both normal mice and mice deafened *via* elimination of their peripheral auditory pathway.

## 5. Conclusions

**Figure 4:**
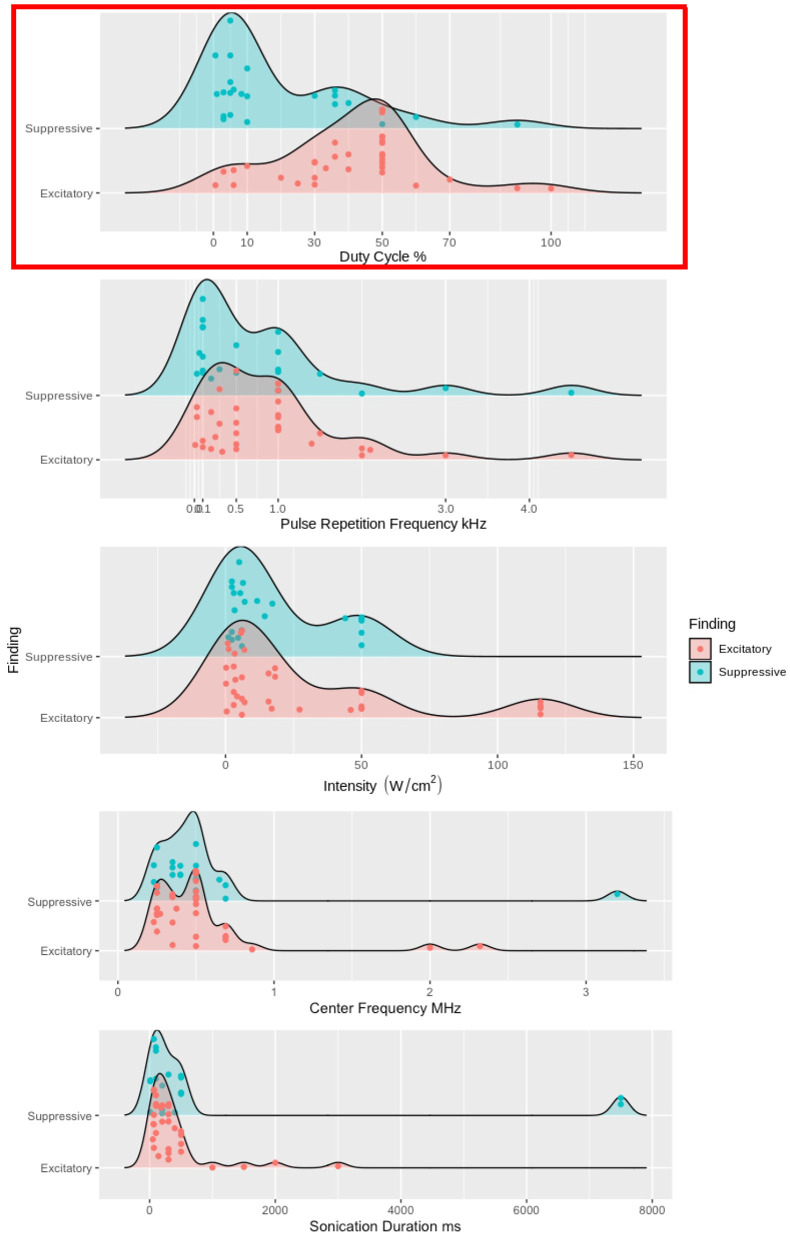
Excitatory and suppressive empirical findings' relationships to DC, PRF, I_SPPA_, f_*c*_, and SD. DC, PRF, I_SPPA_, f_*c*_, and SD are used as grouping factors for excitatory and suppressive findings. We used density plots for each study, but studies with multiple sonication parameters have each one plotted. In the top panel, high DC, above 10%, has the vast majority of the excitatory findings. While ow DC, less than 10%, contains the majority of the suppressive findings, there are still approximately 30% of the suppressive findings above 10% DC. The top panel is highlighted in red because DC is the one sonication parameter that has any distinction between excitatory and suppressive findings. In the four bottom panels, PRF, I_SPPA_, f_*c*_, and SD has no clear distinction between excitatory and suppressive findings.

Pioneering studies of neuromodulatory effects on the central (Fry et al., 1958) and peripheral (Gavrilov et al., 1976) nervous systems established the foundation for the expanding collection of recent US neuromodulation studies. The majority of the findings discussed in this review were excitatory (Table 1) or suppressive (Table 2) and used neuroimaging, electrophysiological recordings, and/or behavioral results (Tufail et al., 2010; Yoo et al., 2011a; Li et al., 2019; Kubanek et al., 2020) to bolster the claims of respective effects. The studies with excitatory and suppressive findings allowed for the development of theoretical models for US's modulation. The modified NICE model predicts that DC will determine the polarity of the neuromodulation independent of other sonication parameters; Figure 4). All of the studies reporting excitatory effects (except 3, Yu et al., 2021; Yuan et al., 2020; Liu et al., 2021) with pulsed US used a higher DC (i.e., above 10%) produced excitatory effects, supporting the modified NICE model. In further support of the modified NICE model, Yoon et al. (2019) tested multiple sonication parameter combinations and found that DC independently determined the polarity of the neuromodulation. However, when using continuous US (i.e., DC = 100%), they found suppressive effects. Despite support for the modified NICE model for US paradigms using higher DC, the majority of studies using a low DC within the suppressive range did not produce suppressive effects (Legon et al., 2014, 2018a,b; Yu et al., 2021) and some studies found suppressive effects using higher DC (Legon et al., 2014, 2018a,b; Darrow et al., 2019; Yu et al., 2021; Yang et al., 2021). Taken together, these empirical findings do not corroborate the modified NICE model's parameter space predictions for suppressive effects.

One possible mechanism of action is of thermal effects from higher ranges of intensity in LIFU (Darrow et al., 2019). However, this finding is in a single animal and is currently debated (Darrow et al., 2020; Spivak et al., 2020). Another possible mechanism is the electrophysiological-mechanical coupling in the neuronal membrane (Jerusalem et al., 2019), which considers mechanosensitive ion channels (Figure 2D, column 3) and membrane conformational states (Figure 2A, column 3). Mechanosensitive ion channels allow for the direct transduction of mechanical energy (e.g., US acoustic wave) into neural signals. These neural signals could also emanate from calcium influx in *hs*TRPA1-expressing mammalian human embryonic kidney-293T cells, which interact with the actin cytoskeleton inducing the calcium influx (Duque et al., 2022). The membrane conformational states have three general frameworks of voltage-induced changes: (1) membrane tension (Figure 2, (2) direct flexoelectricity (Figure 2C), and (3) thermodynamic waves (Figure 2B).

An alternative explanation for these higher DC suppressive effects could be a disruption of thermodynamic waves during action potentials. Action potential propagation has an electrophysiological-coupling that can be modeled by a thermodynamic wave and US can interfere with those thermodynamic waves *via* the pressure wave generated from the acoustic force traveling throughout the neurons at the sonication targeted region. Finally, it is also possible that mechanosensitive ion channels (Figure 2D, column 3) in glial system were preferentially activated in these studies (Ostrow et al., 2011).

The electrophysiological-mechanical coupling in the neuronal membrane and the modified NICE model provide explanations for possible mechanisms of action during or shortly after a US pulse. The longer-lasting effects (e.g., reduction of SEP responses up to 35 min Yoo et al., 2011a and reduction of fMRI BOLD response up to 2 h Chu et al., 2015) along with changes in connectivity (Folloni et al., 2019; Verhagen et al., 2019; Sanguinetti et al., 2020) require additional explanatory mechanisms. These effects could be elicited from cortical plasticity mechanisms of long-term potentiation (LTP) and/or depression (LTD) as proposed for other neurostimulation methods (e.g., TMS Stagg and Nitsche, 2011 or TES Ridding and Ziemann, 2010). Alternatively, repeated or long exposure from US could leave lasting changes on the membrane conformational states due to stored conformation/geometric changes like a cellular conformational memory akin to a change in membrane capacitance following repeated electrical stimulation (Jerusalem et al., 2019). These changes would affect the electrophysiological-mechanical coupling in the neuronal membrane from direct flexoelectricity and/or thermodynamic waves due to changes in dipole configurations and/or changes to thermodynamic properties affecting the soliton wave (Heimburg, 2012).

The neuromodulatory effects of LIFU could be affected by multiple aspects of general anesthesia. General anesthesia has multiple state changes including loss of consciousness, immobility, analgesia, and amnesia. A mechanistic account of anesthesia must account for these state changes. While the exact mechanism of action of anesthesia are unknown and may differ depending on the agent used, the known protein binding sites of certain agents (e.g., propofol) could induce state dependent effects from the modulation of neural activity *via* GABA_A_ (Yip et al., 2013) or nicotinic acetylcholine receptors (Jayakar et al., 2013). These state dependent effects could reduce or increase the neuromodulatory effects of LIFU. Additionally, there are possible alterations to the equilibrium of gel and liquid phases of lipids affecting the ability of channels opening that are dependent on a liquid crystalline state (Lee, 1976; Tsien, 1989). These phase changes could affect the production or annihilation of thermodynamic waves produced by US stimulation due to the phase dependence of the lipids for the soliton model. Finally, noble gas anesthetic agents may produce bubbles in ion channels due to hydrophobic regions in the channel wall allows for cohesive forces of a fluid to pull away from the wall and the bubbles are localized by the rings of non-polar amino acids. This is a more general phenomenon known as capillary evaporation (Roth et al., 2008; Rusanov, 2015). These bubbles form and fill with water and ions switching the channel from conducting to non-conducting while the bubble is present, but when the bubble breaks they become conducting when the water and ions flood the channel (Roth et al., 2008). This bubble formation could be a parallel phenomenon with sonoporation, but acting on a different part of the membrane (i.e., the ion channel instead lipid bilayer) (Jerusalem et al., 2019). These possible mechanisms of action could each influence effects of US neuromodulation, which has not been accounted for in the studies reviewed, but should be in future studies.

In addition to these proposed mechanisms, personalized tuning of parameters needs to be explored and modeled *via* simulations prior to stimulation due to differences in skull thickness/ morphometry, age, brain region targeted, and neural trauma, not to mention differences across species. Skull thickness directly affects the amount mechanical energy reaching the underlying targeted tissue due to mode conversion, reflection, scattering, and bone absorption (Fry and Barger, 1978; Pinton et al., 2012; Phipps et al., 2019). These energy attenuations are typically mitigated by positioning the transducer adjacent to thin parts of the skull (e.g., temporal window). Additionally, the amount of mechanical energy transduced by the neural tissue can be changed by the elasticity of the neural tissue, which is affected by age, cell type, trauma, and density of mechanosensitive ion channels. These important participant inhomogeneities need to be accounted for as our understanding of the mechanisms of action for neuromodulation becomes more refined.

The underlying mechanisms of neuromodulation are rapidly developing and, as more studies are produced, the effects of sonication parameters will be able to be more discretely characterized. The modified NICE model has provided a key link to the relationship between DC and sonoporation. This relationship holds for the excitatory findings explored in this review, but fails to account for the suppressive effects and does not offer a good explanation for the other findings. In this review, the modified NICE model was the only model providing predictions about sonication parameters affecting the underlying mechanisms of neuromodulation. Membrane conformational states, direct flexoelectricity, and thermodynamic waves are also possible mechanisms of LIFU neuromodulation, but need further development to understand how these mechanisms are affected by sonication parameters. These mechanisms need to be further explored for the role of changing the sonication parameters in the amount of mechanical energy delivered to targeted neurons. These changes in mechanical energy will ultimately change the electrophysiological-mechanical coupling. While this review highlighted the modified NICE model, it is not conclusive as the only mechanism for neuromodulation. As the understanding of the mechanisms of LIFU neuromodulation matures, researchers will be able to more precisely tune their sonication parameters to improve the effectiveness of LIFU.

## Author Contributions

JD'I and NR researched the data for the manuscript and prepared the first draft of the manuscript. JS, MM, and AB revised the manuscript for submission. All authors contributed to the article and approved the submitted version.

## Conflict of Interest

AB is founder and major stockholder of Brainsonix Corp. and is a patent holder in the field of FUS brainstimulation. The remaining authors declare that the research was conducted in the absence of any commercial or financial relationships that could be construed as a potential conflict of interest.

## Publisher's Note

All claims expressed in this article are solely those of the authors and do not necessarily represent those of their affiliated organizations, or those of the publisher, the editors and the reviewers. Any product that may be evaluated in this article, or claim that may be made by its manufacturer, is not guaranteed or endorsed by the publisher.
